# Induction of cortical plasticity and improved motor performance following unilateral and bilateral transcranial direct current stimulation of the primary motor cortex

**DOI:** 10.1186/1471-2202-14-64

**Published:** 2013-07-01

**Authors:** Dawson J Kidgell, Alicia M Goodwill, Ashlyn K Frazer, Robin M Daly

**Affiliations:** 1 Centre for Physical Activity and Nutrition Research, Deakin University, Melbourne, Australia

**Keywords:** Transcranial Direct Current Stimulation, Motor Cortex Plasticity, Motor Performance

## Abstract

**Background:**

Transcranial direct current stimulation (tDCS) is a non-invasive technique that modulates the excitability of neurons within the primary motor cortex (M1). Research shows that anodal-tDCS applied over the non-dominant M1 (i.e. unilateral stimulation) improves motor function of the non-dominant hand. Similarly, previous studies also show that applying cathodal tDCS over the dominant M1 improves motor function of the non-dominant hand, presumably by reducing interhemispheric inhibition. In the present study, one condition involved anodal-tDCS over the non-dominant M1 (unilateral stimulation) whilst a second condition involved applying cathodal-tDCS over the dominant M1 and anodal-tDCS over non-dominant M1 (bilateral stimulation) to determine if unilateral or bilateral stimulation differentially modulates motor function of the non-dominant hand. Using a randomized, cross-over design, 11 right-handed participants underwent three stimulation conditions: 1) unilateral stimulation, that involved anodal-tDCS applied over the non-dominant M1, 2) bilateral stimulation, whereby anodal-tDCS was applied over the non-dominant M1, and cathodal-tDCS over the dominant M1, and 3) sham stimulation. Transcranial magnetic stimulation (TMS) was performed before, immediately after, 30 and 60 minutes after stimulation to elucidate the neural mechanisms underlying any potential after-effects on motor performance. Motor function was evaluated by the Purdue pegboard test.

**Results:**

There were significant improvements in motor function following unilateral and bilateral stimulation when compared to sham stimulation at all-time points (all P < 0.05); however there was no difference across time points between unilateral and bilateral stimulation. There was also a similar significant increase in corticomotor excitability with both unilateral and bilateral stimulation immediately post, 30 minutes and 60 minutes compared to sham stimulation (all P < 0.05). Unilateral and bilateral stimulation reduced short-interval intracortical inhibition (SICI) immediately post and at 30 minutes (all P < 0.05), but returned to baseline in both conditions at 60 minutes. There was no difference between unilateral and bilateral stimulation for SICI (P > 0.05). Furthermore, changes in corticomotor plasticity were not related to changes in motor performance.

**Conclusion:**

These results indicate that tDCS induced behavioural changes in the non-dominant hand as a consequence of mechanisms associated with use-dependant cortical plasticity that is independent of the electrode arrangement.

## Background

In recent years, there has been an effort to optimize motor training approaches following neurological injury that employ techniques that non-invasively modulate the excitability of neuronal circuits within the primary motor cortex (M1). In particular, transcranial direct current stimulation (tDCS) of the M1 has emerged as a popular neuromodulation technique, with recent evidence demonstrating modifications in cortical and motor function following stimulation [1]. tDCS is a non-invasive, painless, easy to administer and cost effective procedure with minimal side effects, and thus is ideally suited for use in neuro rehabilitation [2].

A key feature of tDCS is that it adjusts regional brain activity by modifying the membrane potential of neurons [3,4]. The application of low level direct electrical current is delivered through saline soaked electrodes secured above the area of interest on the M1, at intensities of approximately 1–2 mA for periods of 2–20 minutes, with the electrode montage (anodal or cathodal) determining the physiological effect of stimulation (for a detailed review refer to 1). In general, anodal-tDCS of long duration (i.e. up to 20 minutes) induces facilitator effects of motor-evoked potentials (MEPs) [5-7], whilst cathodal-tDCS leads to inhibitory effects [4,8-10]. Single session anodal-tDCS with current intensities of 0.6 mA up to 2 mA applied for between 5–20 minutes has been shown to increase corticomotor excitability for up to 60 minutes after stimulation [2]. Several transcranial magnetic stimulation (TMS) studies have reported increased corticomotor excitability [4,6], reduced intracortical inhibition [11,12] and reduced interhemispheric inhibition (IHI) following both unilateral (anodal-tDCS over the M1) and bilateral tDCS (simultaneously applying cathodal-tDCS over the dominant M1 and anodal-tDCS over non-dominant M1) [13]. However, few studies have compared the effects of unilateral-anodal and bilateral tDCS on modulating motor function [13,14].

The temporary modification in cortical plasticity following anodal-tDCS has been reported to correspond with transient improvements in motor function [11,15]. For example, anodal-tDCS applied over M1 has been shown to improve sequential finger movement tasks, visuomotor coordination, and reaction time and hand function [16-19]. In addition, applying anodal-tDCS to the non-dominant M1 also facilitates motor function in the non-dominant upper limb of healthy adults and stroke affected patients [11,15,20].

Emerging evidence suggests that bilateral tDCS may facilitate corticomotor excitability and motor function to a greater extent than just unilateral anodal-tDCS [13,14]. In healthy adults, 20–40 minutes of bilateral stimulation, with the anode fixed over the non-dominant M1 and the cathode over the dominant M1, facilitates motor function to a greater magnitude compared to previous studies employing unilateral and sham stimulation [13,19,21]. Asymmetric use of the non-dominant hand compared to the dominant hand is associated with reduced motor task performance [22]. Similarly, asymmetries in motor function between the non-dominant and dominant limbs, is a likely consequence of hemispheric differences in corticomotor excitability and inhibition [23-26]. As such, the use of bilateral tDCS could be used to optimize corticomotor excitability in the non-dominant M1 and by default improve motor function.

The potential mechanisms underpinning improvements in motor function following bilateral stimulation, although speculative (and not well understood), may reside in reduced IHI [13,27]. It is hypothesized that during bilateral stimulation, corticomotor excitability of the dominant M1 is reduced with cathodal tDCS, which dampens the inhibitory projections from the dominant onto the non-dominant M1, presumably releasing the non-dominant M1 from inhibition and augmenting the excitatory effect of the anode and seemingly enhances motor function in the non-dominant limb [13,17,19,21].Despite this evidence, there is limited data available to show an association between changes in tDCS induced corticomotor plasticity and improvements in motor function [11,15,20,28]. Although the after-effects of tDCS in inducing corticomotor plasticity are well described [1], there have been no reports of motor function effects in healthy young adults lasting longer than 30 minutes following bilateral tDCS on enhancing motor function. Therefore, the purpose of this study was to determine whether unilateral or bilateral stimulation differentially induces enhanced motor function of the non-dominant hand in healthy adults and to further elucidate the neural mechanisms underlying any potential after-effects on motor function following unilateral and bilateral stimulation.

## Methods

### Participants

Eleven healthy adults aged 22–36 years without a history of upper limb injury or neurological disorder participated in the study. All participants were right hand dominant (mean laterality quotient, 72.5 ± 14.5) according to the 10-item version of the Edinburgh Handedness Inventory [29]. Prior to the experiment, all participants completed the adult safety screening questionnaire to determine their suitability for TMS and tDCS application [30]. All participants gave written informed consent prior to participation in the study, which was approved by the Deakin University Human Research Ethics Committee. All experiments were conducted according to the standards established by the Declaration of Helsinki.

### Experimental approach

**Figure 1:**
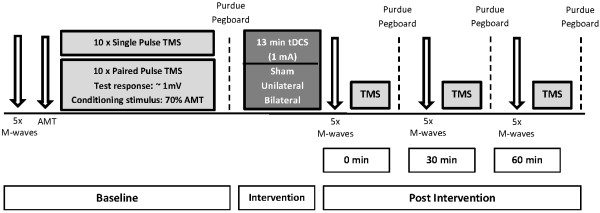
**Schematic representation of the experimental protocol with measures obtained before, immediately after (0 min), 30 and 60 minutes after tDCS.** Time-course measures included assessments of M_MAX_, active motor threshold (AMT), cortical excitability (130% AMT), short-interval intracortical inhibition (SICI) and motor function (pegboard).

Using a randomized, cross-over design; each participant was exposed to 13 minutes of sham, unilateral-anodal or bilateral tDCS applied at 1.0 mA (25 cm^2^ electrodes, current density 0.040 mA/cm^2^). In all tDCS conditions, the anode was placed over the “hot spot” of the non-dominant extensor carpi radialis longus (ECRL) muscle as determined by TMS. The order of these conditions were counterbalanced and randomized across participants, with a one weeks rest between each condition. A purpose made Excel macro was used to randomize each experimental condition. This was a double-blinded trial as the investigator performing the experimental treatment and evaluation along with the participant, was not aware of which tDCS condition was being applied. This was achieved as the tDCS machine used, allowed for the use of a code to determine whether tDCS was active or inactive (sham). Within the sham condition, 50% of the unilateral stimulation and 50% of the bilateral stimulation was randomized for sham stimulation. Single and paired-pulse TMS was used to assess the after-effects of unilateral, bilateral or sham stimulation on corticomotor excitability of the right M1 and motor function of the non-dominant left ECRL. Ten single-pulse (130% of active motor threshold [AMT]), 10 paired-pulse (70% of AMT) and 10 test (test-intensity set to produce MEPs of ~1 mV) TMS stimuli were applied over the cortical area for the left ECRL at baseline, immediately following, 30 and 60 minutes post tDCS, with the order of TMS stimuli (single, paired-pulse or test) prior to and following tDCS, randomized throughout the trials (30 trials in total for each time point). Motor function was measured at each of these time points in all conditions by having participants complete a Purdue pegboard test with their left hand only. Figure 1 displays a schematic representation of the experimental protocol with measures obtained before and after tDCS. Importantly, all electrophysiological measures for each time point were measured prior to the performance of the pegboard, as post MEP facilitation and the effectiveness of SICI has been shown to be modulated immediately following the completion of the pegboard test [25].

### Motor function

The Purdue pegboard was used to assess manual dexterity of the participants’ non-dominant left hand. The task involved picking up small pegs from a well and placing them as quickly as possible in a vertical array of holes using only the index finger and thumb. Motor function was quantified by recording the time taken to complete the left hand side of the peg board. Each participant was tested three times at each time point with the average time recorded.

### Transcranial direct current stimulation of primary motor cortex

Sham, unilateral and bilateral stimulation was delivered by a battery driven constant-current transcranial direct current stimulator (NeuroConn, Ilmenau, Germany) via a pair of conductive rubber electrodes, each positioned inside a saline-soaked surface sponge electrode (25 cm^2^). During unilateral stimulation, the anode was fixed with two straps over the optimal cortical representation of the left ECRL muscle as identified by TMS over the right cortex, and the cathode (25 cm^2^) was placed over the left contralateral supra orbital area. During bilateral stimulation, the anode was placed over the cortical representation of the left ECRL and the cathode was placed over the cortical representation of the right ECRL. For the sham condition, both unilateral and bilateral (50% each) sham stimulation was applied according to the electrode positioning above. In order to obtain the participants perception of discomfort throughout the tDCS conditions, discomfort was assessed using a visual analogy scale (VAS), with “no discomfort” at one end of a 100-mm line and “extremely uncomfortable” at the other, during the first three minutes of cortical stimulation.

### Transcranial magnetic stimulation and electromyography

Focal TMS was used to measure corticomotor excitability and SICI of the contralateral ECRL. Specifically, TMS was applied over the right M1 using a BiStim unit attached to two Magstim. 200^2^ stimulators (Magstim Co, Dyfed, UK) to produce MEPs in the non-dominant left ECRL. A figure-eight coil, with an external loop diameter of 9 cm, was held over the right M1 at the optimum scalp position to elicit MEPs in the left ECRL. The induced current in the brain flowed in a posterior-to-anterior direction. Sites near the estimated centre of the ECRL were explored to determine the optimal site at which the largest MEP amplitude was obtained, and this area was marked by a small ‘x’ in permanent marker. To ensure consistency throughout the study period and reliability of coil placement, the participant and researcher maintained the mark between experimental conditions. Care was taken by the researcher to ensure that the coil was held over the same position on the scalp so that the same area of the M1 was stimulated for all experimental conditions. All TMS measures were taken during weak voluntary contraction, by having the participant hold their hand in line with their wrist (neutral position). Root mean square (rms) electromyography (EMG) of the ECRL was obtained prior to each TMS stimulus to ensure that there were no changes in pre-stimulus rmsEMG which may have altered the MEP amplitude. AMT was determined as the minimum stimulus intensity that produced a small MEP (200 *μ*V in 5 out of 10 consecutive trials) during isometric contraction of the ECRL at 5 ± 2% of maximal rmsEMG activity. A constant level of contraction was maintained with reference to an oscilloscope (HAMEG, Mainhausen, Germany) that displayed the rmsEMG signal in front of the participant. The stimulus intensity started at 50% of maximum stimulator output (MSO) and was altered in increments of ± 1% of MSO until the appropriate threshold level was achieved. All MEP amplitudes were evaluated using an in test-stimulus intensity of 130% AMT.

Surface electromyography (sEMG) activity was recorded from the left ECRL muscle using bipolar Ag-AgCl electrodes. These electrodes were placed on the ECRL muscle, with an inter-electrode distance (centre to centre) of 2 cm with a muscle belly-tendon montage. A grounding strap placed around the wrist was used as a common reference for all electrodes. All cables were fastened with tape to prevent movement artifact. The area of electrode placement was shaven to remove fine hair, rubbed with an abrasive skin rasp to remove dead skin, and then cleaned with 70% isopropyl alcohol. The exact sites were marked with a permanent marker by tracing around the electrode, and this was maintained for the entire three week period by both the researcher and participant to ensure consistency of electrode placement relative to the innervation zone. An impedance meter was used to ensure impedance did not exceed 10 k***Ω*** prior to testing. sEMG signals were amplified (x100-1000), band pass filtered (high pass at 13 Hz, low pass at 1000 Hz), digitized online at 2 kHz for 500 ms, recorded and analyzed using Power Lab 4/35 (AD Instruments, Bella Vista, Australia).

### Short-Interval Intracortical Inhibition (SICI)

The protocol for SICI included 10 unconditioned stimuli, with a test intensity set to produce MEPs of ~1 mV in the ECRL and 10 conditioned stimuli, with a conditioning stimulus intensity set at 70% of AMT to induce SICI. Both AMT and the test stimulus intensity were adjusted at each time point following the removal of tDCS, if required, to ensure that AMT (% MSO) and the test MEP amplitudes were similar (1 mV) prior to and following tDCS. An inter-stimulus interval (ISI) of 3 ms between the conditioning and test stimulus was used [31]. Single and paired-pulse stimuli were presented according to a predetermined randomization protocol, with a 6–9 second time period between each stimulus.

### Maximal compound muscle action potential

Direct muscle responses were obtained from the left ECRL muscle by supramaximal electrical stimulation (pulse width 1 ms) of the brachial plexus (Erbs point) under resting conditions (DS7A, Digitimer, UK). The site of stimulation that produced the largest M-wave was located by positioning the bipolar electrodes in the supraclavicular fossa. An increase in current strength was applied to the brachial plexus until there was no further increase observed in the amplitude of the sEMG response (M_MAX_). To ensure maximal responses, the current was increased an additional 20% and the average M_MAX_ was obtained from five stimuli, with a period of 6–9 seconds separating each stimulus. M_MAX_ was recorded at baseline and at each time point following the removal of tDCS for each condition, to ensure that there were no changes in peripheral muscle excitability that could influence MEP amplitude.

### Data analyses

Pre-stimulus rmsEMG activity was determined in the ECRL 100 ms prior to each TMS stimulus during each condition. Any pre-stimulus rmsEMG that exceeded 5 ± 2% maximal rmsEMG were discarded and the trial repeated. The peak-to-peak amplitude of MEPs evoked as a result of stimulation was measured in the ECRL muscle contralateral to the cortex being stimulated in the period 10–50 ms after stimulation. MEP amplitudes were analyzed using LabChart 7.3.6 software (ADInstruments, Bella Vista, NSW, Australia) after each stimulus was automatically flagged with a cursor, providing peak-to-peak values in mV and were then normalized to M_MAX_. Average MEP amplitudes were obtained for each trial for single, paired-pulse and test TMS for each stimulation block (30 trials for each time point) separately. SICI was quantified by dividing the average paired-pulse MEP by the average single-pulse MEP (test-intensity set to produce MEPs of 1 mV) and multiplying by 100.

### Statistical analysis

A split-plot in time, repeated measures ANOVA was used to compare the effect of each tDCS condition (sham, unilateral and bilateral) on corticomotor plasticity and motor function. When appropriate, univariate and post-hoc (LSD) analyses for pair wise comparisons of means for each dependent measure were used when significant interactions were found. Pearson’s correlations (r) were calculated to assess the association between M1 plasticity and motor performance. For all tests, the Huynh-Feldt correction was applied if the assumption of sphericity was violated. Alpha was set at P ≤ 0.05, and all results are displayed as means ± SEM.

## Results

### Baseline characteristics

There were no differences in M_MAX_, MEP amplitude, SICI, rmsEMG and motor function between conditions at baseline (all P > 0.05; Table 1).

**Table 1:** Mean ± SEM participant characteristics and baseline excitability measures before tDCS

	**Sham**	**Unilateral**	**Bilateral**	***P*-value**
AMT (%)	30.36±	30.36±	29.36±	0.89
	1.71	1.67	1.60	
SI (130% AMT)	39.09±	39.45±	38.38±	0.94
	2.37	2.27	2.16	
SI 1mV (%)	40.09±	38.45±	37.36±	0.76
	2.78	2.56	2.32	
CS (%)	23.00±	23.09±	22.18±	0.86
	1.35	1.28	1.26	
M_MAX_ (mV)	10.09±	10.24±	10.13±	0.99
	1.62	1.61	1.50	
MEP (%M_MAX_)	14.80±	14.13±	13.99±	0.98
	3.28	3.28	3.36	
SICI ratio	29.66±	28.62±	30.07±	0.93
	2.95	2.71	2.73	
rmsEMG	0.056±	0.060±	0.058±	0.90
(130% AMT)	0.005	0.006	0.006	
VAS	1.29±	1.10±	1.64±	0.25
	0.19	0.22	0.27	
Purdue	56.84±	56.23±	51.04±	0.25
Pegboard	40.80	2.89	3.13	0.069

Values are mean ± SEM. AMT, Active motor threshold; CS, conditioning stimulus; SI stimulation intensity to produce 1mV, M_MAX,_ maximum, M-wave; VAS, visual analogue scale.

### Pre-trigger rmsEMG, M_MAX_andVAS

Averaged over all conditions and time points, the mean pre-trigger rmsEMG was 0.061 ± 0.034 mV. Pre-trigger rmsEMG did not vary between single and paired-pulse trials, and there were no changes over time for any condition and thus no time-by-condition interactions were present. M_Max_ did not change as a function of time or by condition, and there were no time-by-condition interactions present, similarly, the perception of discomfort during tDCS did not vary between conditions (all P> 0.05, Table 1).

### Changes in motor function following tDCS

**Figure 2:**
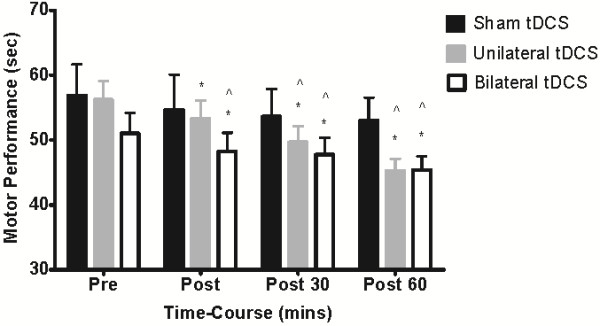
**Mean (± SEM) changes in motor function before and after sham, unilateral and bilateral stimulation.** Motor function improved immediately following unilateral (5%) and bilateral stimulation (5%), with an 11 and 6% improvement at 30 min and a 19 and 10% improvement at 60 min post. *P < 0.05 compared with baseline. ^ P < 0.05 motor function compared with sham stimulation.

Performance in the pegboard task is summarized in Figure 2. Overall, a significant time-by-condition interaction was detected for motor function (F_2, 20_ = 4.119; P = 0.032). Univariate post hoc analyses revealed a 5% increase in motor function immediately following bilateral stimulation when compared to sham stimulation (P < 0.05). Immediately following unilateral stimulation, there was also a significant 5% increase in motor function relative to baseline (P < 0.05), but this change was not significantly different from sham stimulation (time-by-condition interaction, P = 0.274). At 30 minutes post stimulation, the increase in motor function was significant for both unilateral (11%, P < 0.05) and bilateral stimulation (6%, P < 0.05) relative to sham stimulation (F_2, 20_ = 7.426; P = 0.004), but there was no significant difference between these two conditions (P > 0.05). Motor function was still facilitated 60 minutes following unilateral and bilateral stimulation compared to sham stimulation (F_2, 20_ = 10.204; P = 0.001), with unilateral stimulation increasing motor function by 19% (P < 0.05) and bilateral stimulation increasing motor function by 10% (P < 0.05). However, again there were no differences between unilateral and bilateral stimulation (P < 0.05).

### Changes in corticomotor excitability following tDCS

**Figure 3:**
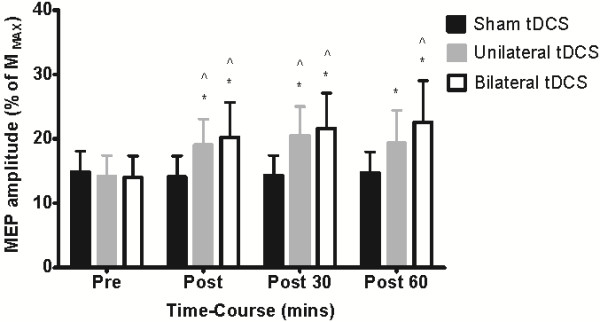
**ECRL MEP amplitude obtained before and after sham, unilateral and bilateral stimulation.** MEP amplitude (Mean ± SEM % M_MAX_) increased immediately following (39 and 40%), 30 minutes (49 and 62%) and 60 minutes post stimulation (33 and 62%). *P < 0.05 compared with baseline; ^ P < 0.05 MEP compared with sham stimulation.

**Figure 4:**
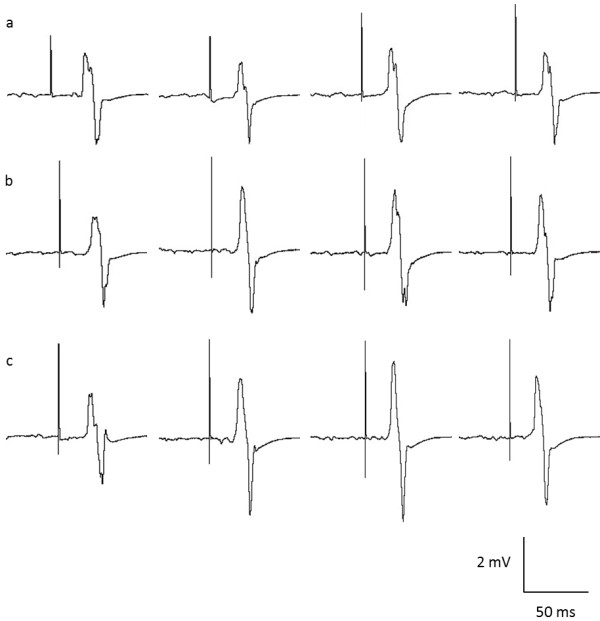
MEP amplitude (130% AMT) sweeps recorded for one participant at baseline, immediately post, 30 minutes and 60 minutes post stimulation for sham (a) unilateral (b) and bilateral (c) stimulation.

Figure 3 shows the mean MEP peak-to-peak amplitude normalized as a percentage of M_MAX_ at each time point for each tDCS condition and Figure 4 shows an example of raw MEP traces (mV) taken from a representative participant at Baseline, post 1, post 2 and post 3 for each condition respectively. Overall, a significant time-by-condition interaction was detected for corticomotor excitability (F_2, 20_ = 5.017; P = 0.017). Univariate post hoc analyses revealed an increase in MEP amplitude immediately following unilateral stimulation (39%, P < 0.05) and bilateral stimulation(40%, P < 0.05) when compared to sham stimulation, but there was no difference between these two conditions at this time point (P = 0.581). Similarly, at 30 minutes post stimulation the magnitude of the increase in MEP amplitude relative to sham stimulation remained significant (F_2, 20_ = 5.711; P = 0.011) for both unilateral stimulation (49%, P < 0.05) and bilateral stimulation (62%, P < 0.05), but again there was no difference between unilateral stimulation and bilateral stimulation (P > 0.05). Interestingly, at 60 minutes post bilateral stimulation, MEP amplitude was still facilitated (62%) compared to sham stimulation (P < 0.05). For unilateral stimulation, there was a significant 33% increase in MEP amplitude at 60 minutes relative to baseline (P < 0.05), but this change was not significantly different from sham stimulation (P > 0.05). Upon conducting correlation analysis, data for the two active tDCS conditions were pooled, as there were no between condition differences in any dependent variable. In the sham stimulation, there were no changes over time in MEPs and as such this condition was excluded from the analysis. The relationship between the change in MEP amplitude and motor function across all time points was not significant (r = 0.152, 0.079, 0.051, all P > 0.05).

### Changes in intracortical inhibition following tDCS

**Figure 5:**
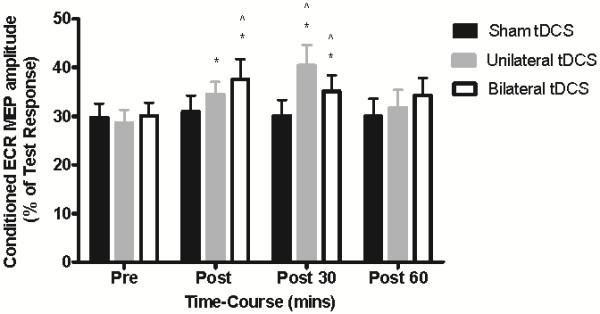
**Mean (± SEM) changes in SICI before and after sham, unilateral and bilateral stimulation.** SICI decreased immediately following unilateral (18%) and bilateral stimulation (25%) with a 45 and 18% improvement at 30 minutes. SICI returned to baseline at 60 minutes in both conditions. *P < 0.05 compared with baseline. ^ P < 0.05 SICI compared with sham stimulation.

The effectiveness of ICI was assessed from the ratio of the MEP size in conditioned and test-alone trials. The effects of tDCS on ICI are summarized in Figure 5. Overall, a significant time-by-condition interaction was detected for SICI (F_2, 20_ = 3.517; P= 0.049). Univariate post hoc analyses revealed significant decrease (25%) in SICI immediately following bilateral stimulation when compared to sham stimulation (P < 0.05). Immediately following unilateral stimulation there was also a significant decrease (18%) in SICI relative to baseline (P < 0.05), but this change was not significantly different from sham stimulation (P > 0.05) or bilateral stimulation (P > 0.05). At 30 minutes post stimulation, there was a decrease in SICI following both unilateral (45%, P < 0.05) and bilateral stimulation (18%, P < 0.05) relative to sham stimulation (F_2, 20_ = 5.295; P = 0.014), but again no significant difference between unilateral stimulation and bilateral stimulation (P > 0.05). At 60 minutes post stimulation there was no difference in SICI between unilateral or bilateral stimulation when compared to sham stimulation (P > 0.05). For both conditions, SICI had returned to baseline levels (time effect: unilateral tDCS, P = 0.289; bilateral tDCS, P = 0.143). Finally, Pearson correlation analysis revealed that the change in SICI by time and condition were not associated with improved motor function (r = 0.103, 0.279 and 0.242).

## Discussion

The main findings from this randomized, cross-over trial was that both unilateral and bilateral tDCS enhanced motor function of the non-dominant hand, which remained facilitated for 60 minutes post stimulation, and induced corticomotor plasticity for up to 60 minutes, but there were no differences in the responses between these two tDCS conditions. Furthermore, there was no association between the change in MEP amplitude and SICI, which are thought to reflect elements of corticomotor plasticity, and the magnitude of motor function for the peg board task in the non-dominant hand of healthy adults.

### tDCS over M1 improves motor function of the non-dominant hand

Several previous studies have reported that tDCS, particularly unilateral stimulation (i.e. anode over non-dominant M1), can improve motor function [16,17,21,32-35], but whether there is a differential effect between unilateral stimulation and bilateral stimulation improving motor function beyond 30 minutes of stimulation is not known. Several previous studies have shown that bilateral stimulation improves motor function immediately after stimulation when compared to unilateral and sham stimulation [13,14], which has been attributed in part to reduction in IHI [14,21]. In contrast to these findings, we found that there was a similar improvement in motor function following both unilateral and bilateral stimulation immediately after, which persisted for up to 60 minutes. This suggests that these two tDCS conditions do not differentially modulate motor function of the non-dominant hemisphere in healthy adults. In part support of these findings, several studies have reported that both unilateral and bilateral stimulation protocols can induce acute improvements in motor function in both upper and lower limb musculature of stroke patients [36-38] and in the elderly [12,20].

The findings that motor function improved following both stimulation conditions is consistent with the results from previous studies, which reported gains in motor function between 9-11% [14,17]. Interestingly, we observed that under both stimulation conditions, motor function was maintained at 30 minutes (11 and 6%) and at 60 minutes (19 and 10%) post stimulation. Furthermore, the time-course improvement of motor function was similar to the time-course effects on corticomotor excitability following tDCS (i.e. increased MEPs) (see 1 for a detailed review). Thus, motor function may have improved as a direct consequence of the effects of tDCS. For instance, an advantage of tDCS is that it modifies the transmembrane potential of neurons, which by default and in most cases, increases M1 excitability and primes a given motor area [14,19,21,27]. Experimental data shows such an effect following unilateral and bilateral stimulation protocols in healthy adults [34], elderly [12] and stroke patients [37,39]. However, the present findings show that motor function is not differentially modulated by the type of tDCS, indicating that the physiological mechanisms potentially regulating motor function are not different. In light of this, we cannot exclude the potential role of other cortical regions, such as the basal ganglia, somatosensory cortex and spinal cord underpinning the improvement in motor function.

### Change in corticomotor excitability following unilateral and bilateral tDCS

An increase in MEP amplitude of the target muscle following unilateral and bilateral tDCS is thought to reflect cortical elements of plasticity [40] via intrinsic changes in excitability of corticospinal cells [41,42]. A change in MEP amplitude that remains elevated for up to 60 minutes has been reported and confirmed by mathematical models, that show tDCS can modify the transmembrane potential [43,44], which influences the excitability of individual neurons.

We hypothesized that unilateral and bilateral stimulation would differentially induce corticomotor excitability, with bilateral stimulation increasing MEP responses compared to unilateral stimulation. Given that unilateral and bilateral stimulation did not differentially induce corticomotor excitability at any time period shows that the same cortical circuits were stimulated to a similar magnitude and that the mechanisms involved in the after-effects for both stimulation conditions were similar. These findings are consistent with a previous unilateral stimulation study, whereby the physiological mechanisms appear to evolve during stimulation that most likely involves modification of the transmembrane potential [43].

There are several reports to show that corticomotor excitability is facilitated for up to 60 and even 90 minutes following unilateral stimulation [1], however there is only one report on the after-effects of bilateral stimulation [27]. Our findings are in agreement with Mordillo-Mateos et al. [27]; however there are some important differences. First, they reported that MEP amplitudes returned to baseline by 20 minutes, whereas we have shown that unilateral and bilateral stimulation increases corticomotor excitability that remains elevated even at 60 minutes post stimulation. Second, we applied tDCS for 13 minutes in all conditions compared to only 5 minutes in their study. A series of studies examining the effects of different durations of unilateral stimulation on corticomotor excitability indicated a linear relationship between the duration of application and the increase in the duration of the after-effects [1,4,6,7,45-49]. Our results confirm these previous findings for unilateral stimulation; however, corticomotor excitability remained elevated at 60 minutes post bilateral stimulation, which shows that bilateral stimulation facilitates corticomotor excitability beyond 20 minutes and that the same after-effects occur as those for unilateral stimulation.

The mechanisms inducing the after-effects of unilateral stimulation are well described [1], however, the mechanism for modulating corticomotor excitability following bilateral stimulation remains uncertain. Given that IHI was not measured in the current study, the induction of corticomotor plasticity via bilateral stimulation may be related to the differences in the induced cortical electrical field current density. Certainly, current flow direction is different when using a motor cortex-contralateral supraorbital arrangement compared to motor cortex-motor cortex arrangement [50]. Recent computational data suggests that the spatial focality of induced cortical electrical field current densities is greater when the active (anode) and return (cathode) electrodes are closer together. For example Faria et al. [50] demonstrated that decreasing the inter-electrode distance resulted in increased current density on the scalp under the edges of the electrode. It’s likely that the reduced inter-electrode distance in the bilateral stimulation configuration has increased the effectiveness of the anodal “target” electrode over the M1 hot spot for the non-dominant ECRL muscle. Therefore, the induction of corticomotor plasticity following bilateral stimulation is likely due to increased focality.

Although only speculative, it’s possible that the cathode decreased corticomotor excitability in the dominant M1 which reduced the inhibitory inputs onto the homologous non-dominant M1, which has subsequently increased corticomotor excitability by increasing the net effect of the anodal stimulation [13,14,21]. One caveat to this interpretation is that we did not measure MEPs for the dominant hemisphere.

### Changes in intracortical inhibition following unilateral and bilateral tDCS

Adjustments in SICI have been reported to be critical in the selective activation of muscles, particularly hand muscles, suggesting that intracortical inhibition is important for motor function [51,52]. No studies have compared the after-effects of unilateral and bilateral tDCS on modulating SICI. Similar to corticomotor excitability, unilateral and bilateral tDCS did not differentially induce SICI. Despite this, SICI was reduced for 30 minutes post stimulation, during both stimulation conditions it returned to baseline by 60 minutes. These findings are consistent with previous studies [7,11], whereby SICI was reduced following unilateral stimulation. However, in contrast to our original hypothesis that bilateral stimulation would reduce SICI further compared to unilateral stimulation, there was no interaction between the type of stimulation and the modulation of GABAergic inhibition. While Nitsche et al., [7] reported that the after-effects of SICI were predominantly induced by unilateral stimulation; a previous study reported no significant difference in SICI with bilateral stimulation [13].

The reduction in intracortical inhibition (for up to 30 minutes) could have contributed to the task-dependent MEP facilitation by unmasking existing excitatory connections to corticospinal neurons activated by TMS [53-55] and/or by enhancing synaptic plasticity at a cortical level [40,41,56]. Taken together, these results demonstrate a noticeable involvement of intracortical synaptic mechanisms that modulate indices of corticomotor plasticity.

We specifically investigated SICI as several plasticity inducing interventions, such as motor skill training, show that reduced SICI is an important mechanism for optimal motor skill learning and for inducing corticomotor plasticity [57]. Additionally, tDCS studies have been advocated to act as a potential primer for improving motor function in healthy adults [16-18,34] and stroke patients [11,16,36,38]. However, the contribution of SICI to improved motor function remains unresolved.

### Association between corticomotor plasticity and motor function from tDCS

There is a widespread belief that increased corticomotor excitability and reduced ICI are associated with the magnitude of behavioral improvement [58]. However, in agreement with several published studies [23,59], we found no association between the changes in corticomotor plasticity (increased MEP amplitude and reduced SICI) and improved motor function (pegboard task). Consistent with these findings, Rogaschet al., [59] reported that increased MEP amplitude and reduced SICI were not associated with the degree of motor learning during a simple index finger motor task in young adults. Interestingly, Williams et al., [13] reported a strong association between changes in MEP amplitude and motor performance, but no association between SICI and motor performance. These mixed findings between indices of corticomotor plasticity and motor function are likely to be the result of many factors that are known to influence the plasticity response, such as the extent of skilled hand use, prior history of synaptic activity, genetic factors (e.g. brain derived neurotrophic factor gene) [60], and gender, with the potential effect of the menstrual cycle [61]. Nonetheless, motor function still improved following unilateral and bilateral stimulation, which provides further evidence that tDCS may be viable rehabilitation tool following neuromuscular injury or disease.

There are several limitations associated with this study. Indices of corticomotor plasticity were only measured from the non-dominant hemisphere, therefore, it is unclear whether cathodal stimulation over the dominant hemisphere would result in reduced corticomotor excitability as previous studies have suggested [1,9]. However, there is evidence that bilateral stimulation can improve motor function of the non-dominant hand, with the reported mechanisms related to modifications in IHI following bilateral stimulation, without any TMS measures. Also, intracortical facilitation (ICF) was not measured, and as such the changes in SICI may have been as a result of increased ICF. Indeed, some evidence shows increased ICF following tDCS [7]. Finally, it could be suggested that measuring MEPs and SICI from the ECRL muscle may lack specificity to the pegboard task that involves intrinsic muscles of the hand. However, the performance of the pegboard task requires the coordinated control and activation of the muscles of the entire arm, and not just the intrinsic muscles of the hand and fingers. Further, there are several kinematic phases involved, with the transportation of the pegs to be placed in the well, governed largely by the muscles proximal to the hand, and as such we don’t believe that recording from the ECR was a distinct limitation.

## Conclusions

Previous studies have demonstrated improved motor function following both unilateral and bilateral stimulation protocols; however few have compared the time course effects of tDCS on modulating motor function. We examined the effects of a single-session of unilateral stimulation, bilateral and sham stimulation on modulating motor function of the non-dominant limb and indices of corticomotor plasticity. In healthy adults, the extent of motor function improvement and corticomotor plasticity were similar between unilateral and bilateral tDCS. Therefore, the physiological mechanisms regulating motor function were not different. Nevertheless, the present data indicate that tDCS induces behavioral changes in the non-dominant hand as a consequence of mechanisms associated with use-dependent cortical plasticity and is not influenced by the tDCS electrode arrangement.

## Competing interest

All authors declare that they have no competing interests.

## Authors’ contributions

DJK.conception and design of research; DJK. performed experiments; AKF analyzed data; DJK, AMG, AKF and RMD interpreted results of experiments; DJK and AKF prepared figures; DJK drafted manuscript; DJK, AMG, AKF and RMD edited and revised manuscript. All authors read and approved the final manuscript.

## References

[B1] Nitsche MA Cohen LG Wassermann EM Priori A Lang N Antal A Paulus W Hummel F Boggio PS Fregni F Pascual-Leone A Transcranial direct current stimulation: State of the art 2008 Brain Stim 2008 1 206 223 10.1016/j.brs.2008.06.004 20633386

[B2] Bastani A Jaberzadeh S Does anodal transcranial direct current stimulation enhance excitability of the motor cortex and motor function in healthy individuals and subjects with stroke: A systematic review and meta-analysis Clin Neurophysiol 2012 123 644 657 10.1016/j.clinph.2011.08.029 21978654

[B3] Liebetanz D Nitsche MA Tergau F Paulus W Pharmacological approach to the mechanisms of transcranial DC-stimulation induced aftereffects of human motor cortex excitability Brain 2002 125 2238 2247 10.1093/brain/awf238 12244081

[B4] Nitsche MA Paulus W Excitability changes induced in the human motor cortex by weak transcranial direct current stimulation J Physiol 2000 527 633 639 10.1111/j.1469-7793.2000.t01-1-00633.x 10990547 PMC2270099

[B5] Lang N Nitsche MA Dileone M Mazzone P De-Andrés-Arés J Diaz-Jara L Paulus W Di-Lazzaro V Oliviero A Transcranial direct current stimulation effects on I-wave activity in humans J Neurophysiol 2011 105 2802 2810 10.1152/jn.00617.2010 21430275

[B6] Nitsche MA Paulus W Sustained excitability elevations induced by transcranial DC motor cortex stimulation in humans Neurol 2001 57 1899 1901 10.1212/WNL.57.10.1899 11723286

[B7] Nitsche MA Seeber A Frommann K Klein C Rochford C Nitsche MS Fricke K Liebetanz D Lang N Antal A Paulus W Tergau F Modulating parameters of excitability during and after transcranial direct current stimulation of the human motor cortex J Physiol 2005 568 291 303 10.1113/jphysiol.2005.092429 16002441 PMC1474757

[B8] Di-Lazzaro V Manganelli F Dileone M Notturno F Esposito M Capasso M Dubbioso R Pace M Ranieri F Minicuci G Santoro L Uncini A The effects of prolonged cathodal direct current stimulation on the excitatory and inhibitory circuits of the ipsilateral and contralateral motor cortex J Neural Transm 2012 119 1499 1506 10.1007/s00702-012-0845-4 22711234

[B9] McCambridge AB Bradnam LV Stinear CM Byblow WD Cathodal transcranial direct current stimulation of the primary motor cortex improves selective muscle activation in the ipsilateral arm J Neurophysiol 2011 105 2937 2942 10.1152/jn.00171.2011 21511707

[B10] Nitsche MA Nitsche MS Klein CC Tergau F Rothwell JC Paulus W Level of action of cathodal DC polarisation induced inhibition of the human motor cortex Clin Neurophysiol 2003 114 600 604 10.1016/S1388-2457(02)00412-1 12686268

[B11] Hummel F Celnik P Giraux P Floel A Wu WH Gerloff C Cohen LG Effects of non-invasive cortical stimulation on skilled motor function in chronic stroke Brain 2005 128 490 499 10.1093/brain/awh369 15634731

[B12] Hummel FC Heise K Celnik P Floel A Gerloff C Cohen LG Facilitating skilled right hand motor function in older subjects by anodal polarization over the left primary motor cortex Neurobiol Aging 2010 31 2160 2168 10.1016/j.neurobiolaging.2008.12.008 19201066 PMC2995492

[B13] Williams JA Pascual-Leone A Fregni F Interhemispheric modulation induced by cortical stimulation and motor training PhysTher 2010 90 398 410 10.2522/ptj.20090075 20110339

[B14] Vines BW Cerruti C Schlaug G Dual-hemisphere tDCS facilitates greater improvements for healthy subjects’ non-dominant hand compared to uni-hemisphere stimulation BMC Neurosci 2008 9 103 10.1186/1471-2202-9-103 18957075 PMC2584652

[B15] Fregni F Gimenes R Valle AC Ferreira MJL Rocha RR Natalle L Bravo R Rigonatti SP Freedman SD Nitsche MA Pascual-Leone A Boggio PS A randomized, sham-controlled, proof of principle study of transcranial direct current stimulation for the treatment of pain in fibromyalgia Arthritis Rheum 2006 54 3988 3998 10.1002/art.22195 17133529

[B16] Antal A Nitsche MA Kincses TZ Kruse W Hoffmann KP Paulus W Facilitation of visuo-motor learning by transcranial direct current stimulation of the motor and extrastriate visual areas in humans Eur J Neurosci 2004 19 2888 2892 10.1111/j.1460-9568.2004.03367.x 15147322

[B17] Boggio PS Castro LO Savagim EA Braite R Cruz VC Rocha RR Rigonatti SP Silva MTA Fregni F Enhancement of non-dominant hand motor function by anodal transcranial direct current stimulation NeurosciLett 2006 404 232 236 10.1016/j.neulet.2006.05.051 16808997

[B18] Nitsche MA Schauenburg A Lang N Liebetanz D Exner C Paulus W Tergau F Facilitation of Implicit Motor Learning by Weak Transcranial Direct Current Stimulation of the Primary Motor Cortex in the Human J Cog Neurosci 2003 15 619 626 10.1162/089892903321662994 12803972

[B19] Vines BW Nair DG Schlaug G Contralateral and ipsilateral motor effects after transcranial current stimulation Neuroreport 2006 17 671 674 10.1097/00001756-200604240-00023 16603933

[B20] Zimerman M Heise KF Hoppe J Cohen LG Gerloff C Hummel FC Modulation of training by single-session transcranial direct current stimulation to the intact motor cortex enhances motor skill acquisition of the paretic hand Stroke 2012 43 2185 2191 10.1161/STROKEAHA.111.645382 22618381 PMC4879963

[B21] Vines BW Nair DG Schlaug G Modulating activity in the motor cortex affects performance for the two hands differently depending upon which hemisphere is stimulated Eur J Neurosci 2008 28 1667 1673 10.1111/j.1460-9568.2008.06459.x 18973584

[B22] Armstrong CA Oldham JA A comparison of dominant and non-dominant hand strengths J Hand Surg-Brit Eur 1999 24 421 425 10.1054/jhsb.1999.0236 10473148

[B23] Cirillo J Rogasch N Semmler J Hemispheric differences in use-dependent corticomotor plasticity in young and old adults Exp Brain Res 2010 205 57 68 10.1007/s00221-010-2332-1 20574685

[B24] De-Gennaro L Cristiani R Bertini M Curcio G Ferrara M Fratello F Romei V Rossini PM Handedness is mainly associated with an asymmetry of corticospinal excitability and not of transcallosal inhibition Clin Neurophysiol 2004 115 1305 1312 10.1016/j.clinph.2004.01.014 15134697

[B25] Garry MI Kamen G Nordstrom MA Hemispheric differences in the relationship between corticomotor excitability changes following a fine-motor task and motor learning J Neurophysiol 2004 91 1570 1578 10.1152/jn.00595.2003 14627660

[B26] Semmler JG Nordstrom MA Hemispheric Differences in Motor Cortex Excitability During a Simple Index Finger Abduction Task in Humans J Neurophysiol 1998 79 1246 1254 9497406 10.1152/jn.1998.79.3.1246

[B27] Mordillo-Mateos L Turpin-Fenoll L Millan-Pascual J Nunez-Perez N Panyavin I Gomez-Arguelles JM Botia-Paniagua E Foffani G Lang N Oliviero A Effects of simultaneous bilateral tDCS of the human motor cortex Brain Stim 2011 5 214 222 10.1016/j.brs.2011.05.001 21782545

[B28] Bolognini N Vallar G Casati C Abdul Latif L El-Nazer R Williams J Neurophysiological and behavioural effects of tDCS combined with constraint-induced movement therapy in post stroke patients Neurorehabil Neural Repair 2011 25 819 829 10.1177/1545968311411056 21803933

[B29] Oldfield RC The assessment and analysis of handedness: The Edinburgh inventory Neuropsychologia 1971 9 97 113 10.1016/0028-3932(71)90067-4 5146491

[B30] Keel J Smith M Wassermann E A safety screening questionnaire for transcranial magnetic stimulation Clin Neurophysiol 2001 112 720 10.1016/S1388-2457(00)00518-6 11332408

[B31] Kujirai T Caramia M Rothwell JC Day B Thompson P Ferbert A Wroe S Asselman P Marsden CD Corticocortical inhibition in human motor cortex J Physiol 1993 471 501 519 8120818 10.1113/jphysiol.1993.sp019912 PMC1143973

[B32] Lindenberg R Renga V Zhu L Nair D Schlaug G Bihemisphere brain stimulation facilitates motor recovery in chronic stroke patients Neurol 2010 75 2176 2184 10.1212/WNL.0b013e318202013a PMC3013585 21068427

[B33] Nitsche MA Grundey J Liebetanz D Lang N Tergau F Paulus W Catecholaminergic consolidation of motor cortical neuroplasticity in humans Cereb Cortex 2004 14 1240 1245 10.1093/cercor/bhh085 15142961

[B34] Reis J Schambra HM Cohen LG Buch ER Fritsch B Zarahn E Celnik PA Krakauer JW Noninvasive cortical stimulation enhances motor skill acquisition over multiple days through an effect on consolidation P NatlAcadSci 2009 106 1590 1595 10.1073/pnas.0805413106 PMC2635787 19164589

[B35] Tanaka S Hanakawa T Honda M Watanabe K Enhancement of pinch force in the lower leg by anodal transcranial direct current stimulation Exp Brain Res 2009 196 459 465 10.1007/s00221-009-1863-9 19479243 PMC2700246

[B36] Hummel FC Cohen LG Non-invasive brain stimulation: a new strategy to improve neurorehabilitation after stroke? Lancet Neurol 2006 5 708 712 10.1016/S1474-4422(06)70525-7 16857577

[B37] Lefebvre S Laloux P Peeters A Desfontaines P Jamart J Vandermeeren Y Dual-tDCS enhances online motor skill learning and long-term retention in chronic stroke patients Front Hum Neurosci 2013 6 1 17 10.3389/fnhum.2012.00343 PMC3541043 23316151

[B38] Tanaka S Takeda K Otaka Y Kita K Osu R Honda M Sadato N Hanakawa T Watanabe K Single session of transcranial direct current stimulation transiently increases knee extensor force in patients with hemiparetic stroke Neurorehabil Neural Repair 2011 25 565 569 10.1177/1545968311402091 21436391

[B39] Boggio PS Nunes A Rigonatti SP Nitsche MA Pascual-Leone A Fregni F Repeated sessions of noninvasive brain DC stimulation is associated with motor function improvement in stroke patients Res NeurolNeurosci 2007 25 123 129 17726271

[B40] Ziemann U Muellbacher W Hallett M Cohen LG Modulation of practice-dependent plasticity in human motor cortex Brain 2001 124 1171 1181 10.1093/brain/124.6.1171 11353733

[B41] Classen J Liepert J Wise SP Hallett M Cohen LG Rapid plasticity of human cortical movement representation induced by practice J Neurophysiol 1998 79 1117 1123 9463469 10.1152/jn.1998.79.2.1117

[B42] Stagg CJ Nitsche MA Physiological basis of transcranial direct current stimulation Neuroscientist 2011 17 37 53 10.1177/1073858410386614 21343407

[B43] Miranda PC Lomarev M Hallett M Modeling the current distribution during transcranial direct current stimulation Clin Neurophysiol 2006 117 1623 1629 10.1016/j.clinph.2006.04.009 16762592

[B44] Wagner T Fregni F Fecteau S Grodzinsky A Zahn M Pascual-Leone A Transcranial direct current stimulation: A computer-based human model study NeuroImage 2007 35 1113 1124 10.1016/j.neuroimage.2007.01.027 17337213

[B45] Boros K Poreisz C Munchau A Paulus W Nitsche MA Premotor transcranial direct current stimulation (tDCS) affects primary motor excitability in humans Eur J Neuro 2008 27 1292 1300 10.1111/j.1460-9568.2008.06090.x 18312584

[B46] Fricke K Seeber AA Thirugnanasambandam N Paulus W Nitsche MA Rothwell JC Time course of the induction of homeostatic plasticity generated by repeated transcranial direct current stimulation of the human motor cortex J Neurophysiol 2011 105 1141 1149 10.1152/jn.00608.2009 21177994

[B47] Furubayashi T Terao Y Arai N Okabe S Mochizuki H Hanajima R Hamada M Yugeta A Inomata-Terada S Ugawa Y Short and long duration transcranial direct current stimulation (tDCS) over the human motor area Exp Brain Res 2008 185 279 286 10.1007/s00221-007-1149-z 17940759

[B48] Purpura DP McMurtry JG Intracellular activities and evoked potential changes during polarization of motor cortex J Neurophysiol 1965 28 166 185 14244793 10.1152/jn.1965.28.1.166

[B49] Utz KS Dimova V Oppenländer K Kerkhoff G Electrified minds: Transcranial direct current stimulation (tDCS) and Galvanic Vestibular Stimulation (GVS) as methods of non-invasive brain stimulation in neuropsychology—A review of current data and future implications Neuropsychologia 2010 48 2789 2810 10.1016/j.neuropsychologia.2010.06.002 20542047

[B50] Faria P Hallett M Miranda PC A finite element analysis of the effect of electrode area and inter-electrode distance on the spatial distribution of the current density in tDCS J Neural Eng 2011 8 066017 10.1088/1741-2560/8/6/066017 22086257 PMC3411515

[B51] Stinear CM Byblow WD Role of intracortical inhibition in selective hand muscle activation J Neurophysiol 2003 89 2014 2020 12611950 10.1152/jn.00925.2002

[B52] Zoghi M Pearce SL Nordstrom MA Differential modulation of intracortical inhibition in human motor cortex during selective activation of an intrinsic hand muscle J Physiol 2003 550 933 946 10.1113/jphysiol.2003.042606 12807989 PMC2343069

[B53] Nitsche MA Fricke K Henschke U Schlitterlau A Liebetanz D Lang N Henning S Tergau F Paulus W Pharmacological modulation of cortical excitability shifts induced by transcranial direct current stimulation in humans J Physiol 2003 553 293 301 10.1113/jphysiol.2003.049916 12949224 PMC2343495

[B54] Butefisch CM Davis BC Wise SP Sawaki L Kopylev L Classen J Cohen LG Mechanisms of use-dependent plasticity in the human motor cortex P NatlAcadSci U.S.A 2000 97 3661 3665 10.1073/pnas.97.7.3661 PMC16296 10716702

[B55] Floeter MK Rothwell JC Releasing the brakes before pressing the gas pedal Neurol 1999 53 664 665 10.1212/WNL.53.4.664 10489022

[B56] Pascual-Leone A Nguyet D Cohen LG Brasil-Neto JP Cammarota A Hallett M Modulation of muscle responses evoked by transcranial magnetic stimulation during the acquisition of new fine motor skills J Neurophysiol 1995 74 1037 1045 7500130 10.1152/jn.1995.74.3.1037

[B57] Liepert J Classen J Cohen LG Hallett M Task-dependent changes of intracortical inhibition Exp Brain Res 1998 118 421 426 10.1007/s002210050296 9497149

[B58] Li Voti P Conte A Suppa A Lezzi E Bologna M Aniello M Defazio G Rothwell JC Berardelli A Correlation between cortical plasticity, motor learning and BDNF genotype in healthy subjects Exp Brain Res 2011 212 91 99 10.1007/s00221-011-2700-5 21537966

[B59] Rogasch NC Dartnall TJ Cirillo J Nordstrom MA Semmler JG Corticomotor plasticity and learning of a ballistic thumb training task are diminished in older adults J ApplPhysiol 2009 107 1874 1883 10.1152/japplphysiol.00443.2009 19833810

[B60] Ridding MC Ziemann U Determinants of the induction of cortical plasticity by non-invasive brain stimulation in healthy subjects J Physiol 2010 588 2291 2304 10.1113/jphysiol.2010.190314 20478978 PMC2915507

[B61] Chaeib L Antal A Paulus W Gender-specific modulation of short-term neuroplasticity in the visual cortex induced by transcranial direct current stimulation Vis Neuro 2008 25 77 81 10.1017/S0952523808080097 18282312

